# Supplementary material for: Identification of Novel Antimicrobial Resistance Genes Using Machine Learning, Homology Modeling, and Molecular Docking
Source: Microorganisms. 2022 Oct 23;10(11):2102. doi: 10.3390/microorganisms10112102 (PMC9693463; doi:10.3390/microorganisms10112102)
Supplement: Supplementary file 1 [file microorganisms-10-02102-s001.zip › Supplementary Figure S10.pdf]

Homology Modeling and Molecular Dockings for *S. enterica*

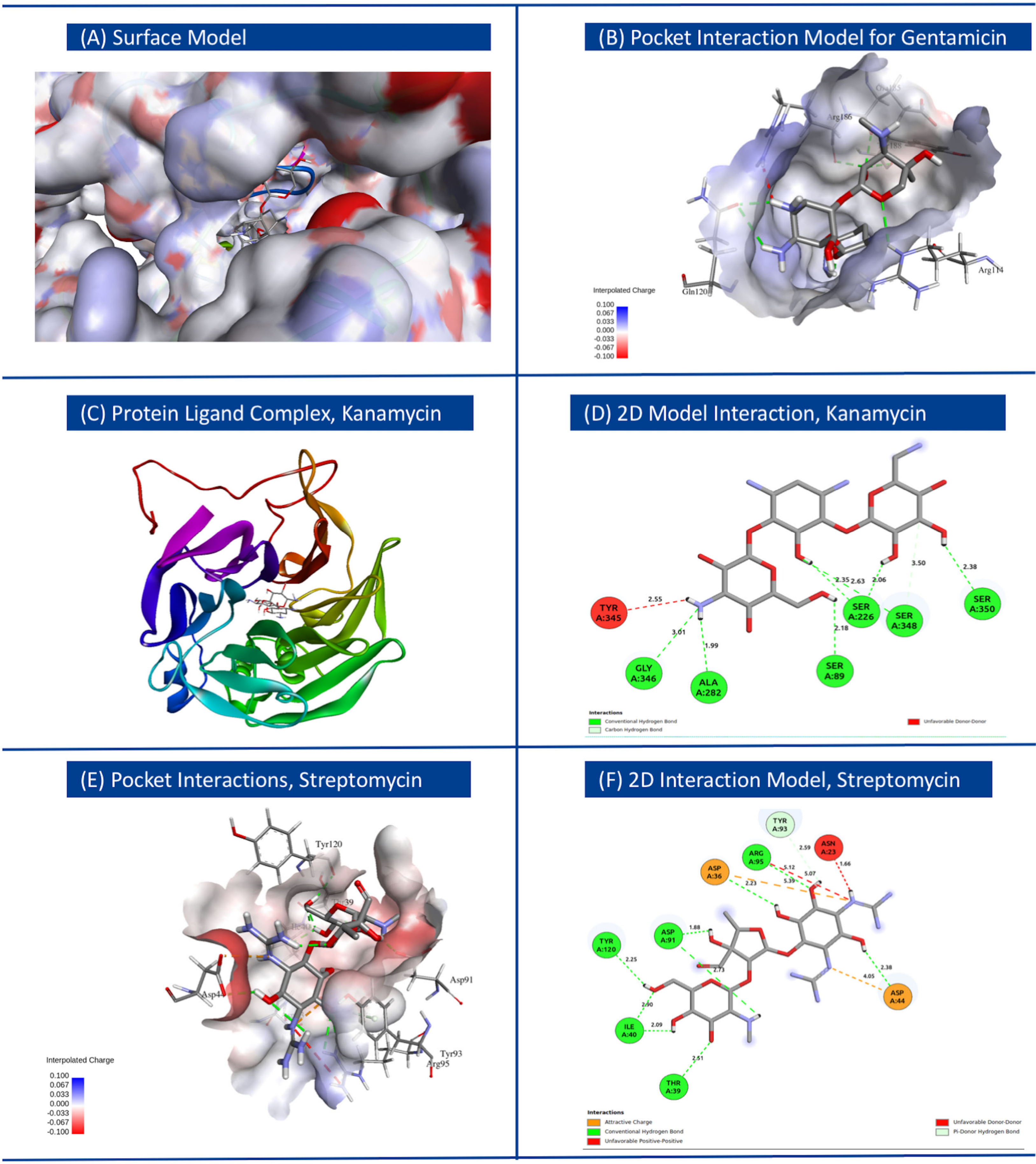

Supplementary Figure 10. Homology modeling docking of novel AMR genes predicted for *S. enterica* for antibiotics (Gentamicin, Kanamycin, and Streptomycin). **10a.** Surface model of receptor (WP_025766410.1). **10b.** Pocket interaction for receptor (WP_025766410.1) and ligand (Gentamicin). **10c.** Receptor (WP_001643958.1) and ligand (Kanamycin) complex. **10d.** 2D model for receptor (WP_001643958.1) **10e.** Pocket interaction for Receptor (WP_000465133.1) and ligand (Streptomycin) **10f.** 2D Interaction Model for receptor (WP_000465133.1) and ligand (Streptomycin).
