# Supplementary material for: Identification of Novel Antimicrobial Resistance Genes Using Machine Learning, Homology Modeling, and Molecular Docking
Source: Microorganisms. 2022 Oct 23;10(11):2102. doi: 10.3390/microorganisms10112102 (PMC9693463; doi:10.3390/microorganisms10112102)
Supplement: Supplementary file 1 [file microorganisms-10-02102-s001.zip › Supplementary Figure S6.pdf]

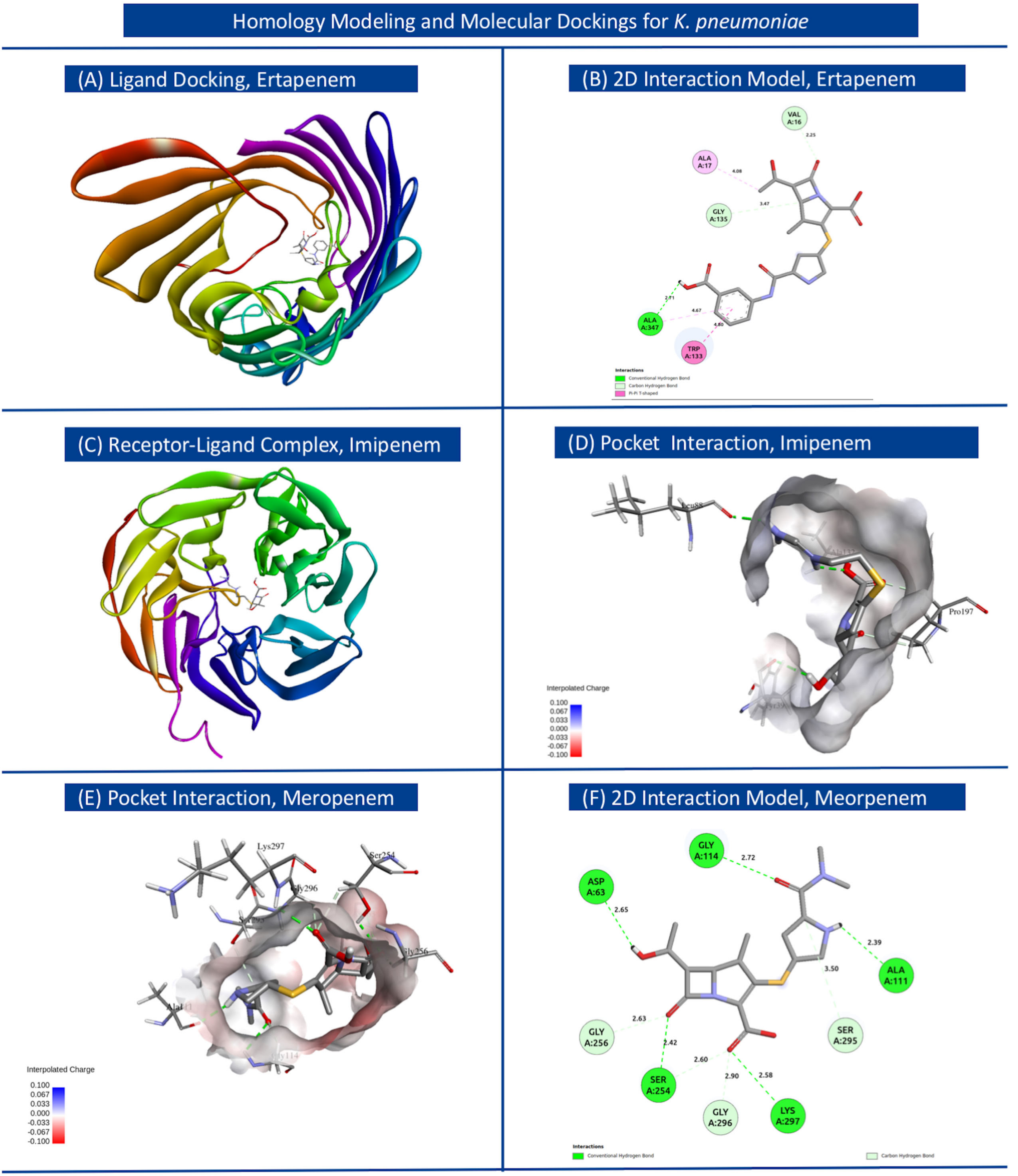

Supplementary Figure 6. Homology modeling docking of novel AMR genes predicted for *K. pneumoniae* for antibiotics (Ertapenem, Imipenem, and Meropenem). **6a.** Receptor (WP_049116479.1) and ligand (Ertapenem) complex. **6b.** 2D interaction model for receptor (WP_049116479.1) and ligand (Ertapenem). **6c.** Receptor (WP_064146913.1) and ligand (Imipenem) complex. **6d.** Pocket interaction for receptor (WP_064146913.1) and ligand (Imipenem). **6e.** Pocket Interaction for receptor (WP_125961907.1) and ligand (Meropenem). **6f** 2D Interaction Model for receptor (WP_125961907.1) and ligand (Meropenem).
