# Supplementary material for: Identification of Novel Antimicrobial Resistance Genes Using Machine Learning, Homology Modeling, and Molecular Docking
Source: Microorganisms. 2022 Oct 23;10(11):2102. doi: 10.3390/microorganisms10112102 (PMC9693463; doi:10.3390/microorganisms10112102)
Supplement: Supplementary file 1 [file microorganisms-10-02102-s001.zip › Supplementary Figure S7.pdf]

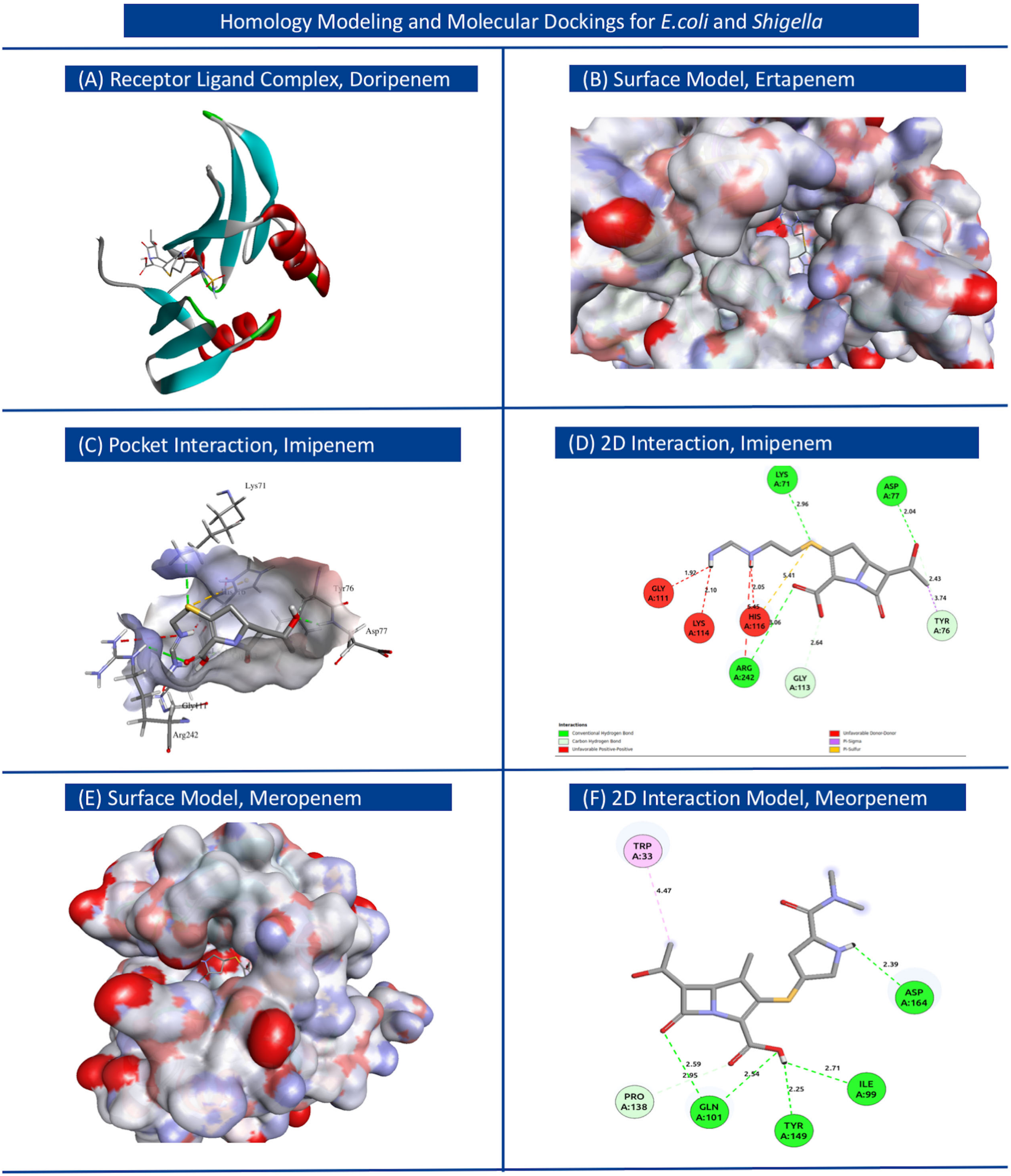

Supplementary Figure 7. Homology modeling docking of novel AMR genes predicted for *E. coli* and *Shigella* for antibiotics (Dorippenem, Ertapenem, Imipenem, and Meropenem). **7a.** Receptor (WP_004201167.1) and ligand (Doripenem) complex. **7b.** Surface model for receptor (WP_001394742.1) and ligand (Ertapenem). **7c.** Pocket interaction of Receptor (WP_004152394.1) and ligand (Imipenem) complex. **7d.** 2D Interaction model for receptor (WP_004152394.1) and ligand (Imipenem). **7e.** Surface model for receptor (WP_032488579.1.1) and ligand (Meropenem). **7f.** 2D Interaction Model for receptor (WP_032488579.1.1) and ligand (Meropenem).
