# Supplementary material for: Identification of Novel Antimicrobial Resistance Genes Using Machine Learning, Homology Modeling, and Molecular Docking
Source: Microorganisms. 2022 Oct 23;10(11):2102. doi: 10.3390/microorganisms10112102 (PMC9693463; doi:10.3390/microorganisms10112102)
Supplement: Supplementary file 1 [file microorganisms-10-02102-s001.zip › Supplementary Figure S8.pdf]

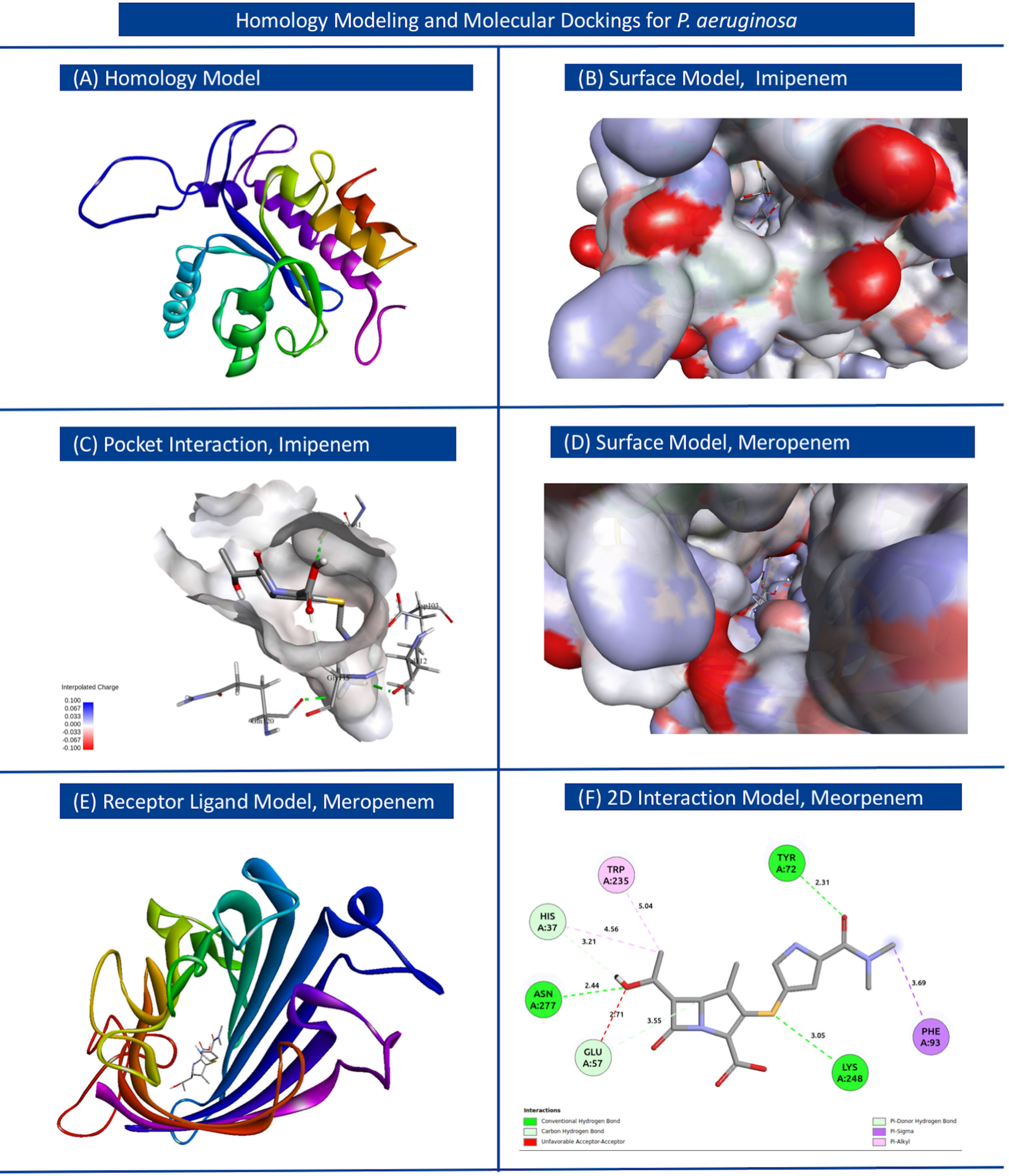

Supplementary Figure 8. Homology modeling docking of novel AMR genes predicted for *P. aeruginosa* for antibiotics (Imipenem and Meropenem). **8a.** Homology model of receptor (WP_031628187.1). **8b.** Surface model for receptor (WP_031628187.1) and ligand (Imipenem). **8c.** Pocket interaction of Receptor (WP_031628187.1) and ligand (Imipenem) complex. **8d.** Surface model for receptor (WP_023097121.1) and ligand (Imipenem). **8e.** Receptor (WP_023097121.1) and ligand (Meropenem) complex. **8f.** 2D Interaction Mode for receptor (WP_023097121.1) and ligand (Meropenem).
