# Supplementary material for: Identification of Novel Antimicrobial Resistance Genes Using Machine Learning, Homology Modeling, and Molecular Docking
Source: Microorganisms. 2022 Oct 23;10(11):2102. doi: 10.3390/microorganisms10112102 (PMC9693463; doi:10.3390/microorganisms10112102)
Supplement: Supplementary file 1 [file microorganisms-10-02102-s001.zip › Supplementary Figure S9.pdf]

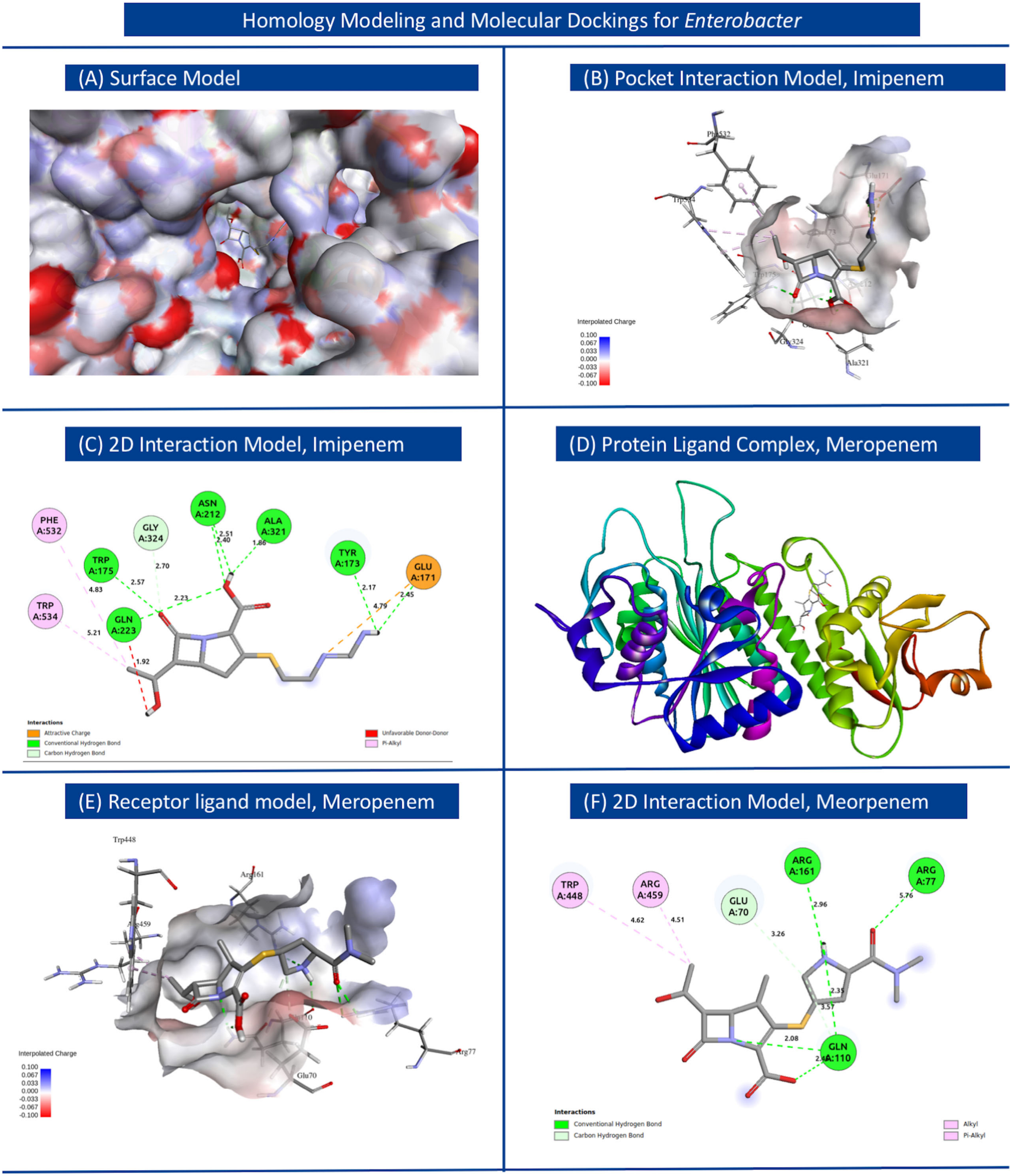

Supplementary Figure 9. Homology modeling docking of novel AMR genes predicted for *Enterobacter* for antibiotics (Imipenem and Meropenem). **9a.** Surface model of receptor (WP_061096807.1). **9b.** Pocket interaction for receptor (WP_061096807.1) and ligand (Imipenem). **9c.** 2D model for Receptor (WP_061096807.1) and ligand (Imipenem) complex. **9d.** Receptor (WP_023337592.1) and ligand (Meropenem) complex. **9e.** Pocket interaction for Receptor (WP_023337592.1) and ligand (Meropenem) **9f.** 2D Interaction Model for receptor (WP_023337592.1) and ligand (Meropenem).
