# Supplementary material for: Identification of Novel Antimicrobial Resistance Genes Using Machine Learning, Homology Modeling, and Molecular Docking
Source: Microorganisms. 2022 Oct 23;10(11):2102. doi: 10.3390/microorganisms10112102 (PMC9693463; doi:10.3390/microorganisms10112102)
Supplement: Supplementary file 1 [file microorganisms-10-02102-s001.zip › Supplementary Table S15.pdf]

**Supplementary Table: S15:** The hyper-parameter details of all the algorithms used in this study. Some algorithms have used default parameters and some custom optimizations, which are also available in source code provided at the GitHub site.

| SN. | Algorithm | Parameters |
|---|---|---|
| 1. | Logistic Regression | max_iter= 10000 |
| 2. | Gaussian NB | default |
| 3. | Support Vector Machine | kernel = 'rbf', probability=True |
| 4. | Decision Tree Classifier | criterion = 'entropy', random_state=1 |
| 5. | Random Forest Classifier | n_estimators = 10, criterion = 'entropy', random_state=0 |
| 6. | K-Neighbors Classifier | n_neighbors = 5, metric = 'minkowski', p = 2 |
| 7. | Linear Discriminant Analysis | Default |
| 8. | Multinomial NB | default |
| 9. | Ada Boost Classifier | default |
| 10. | Gradient Boosting Classifier | default |
| 11 | Extra Trees Classifier | default |
| 12. | Bagging Classifier | default |
