# Supplementary figures and images for: Identification of Novel Antimicrobial Resistance Genes Using Machine Learning, Homology Modeling, and Molecular Docking
Source: Microorganisms. 2022 Oct 23;10(11):2102. doi: 10.3390/microorganisms10112102 (PMC9693463; doi:10.3390/microorganisms10112102)

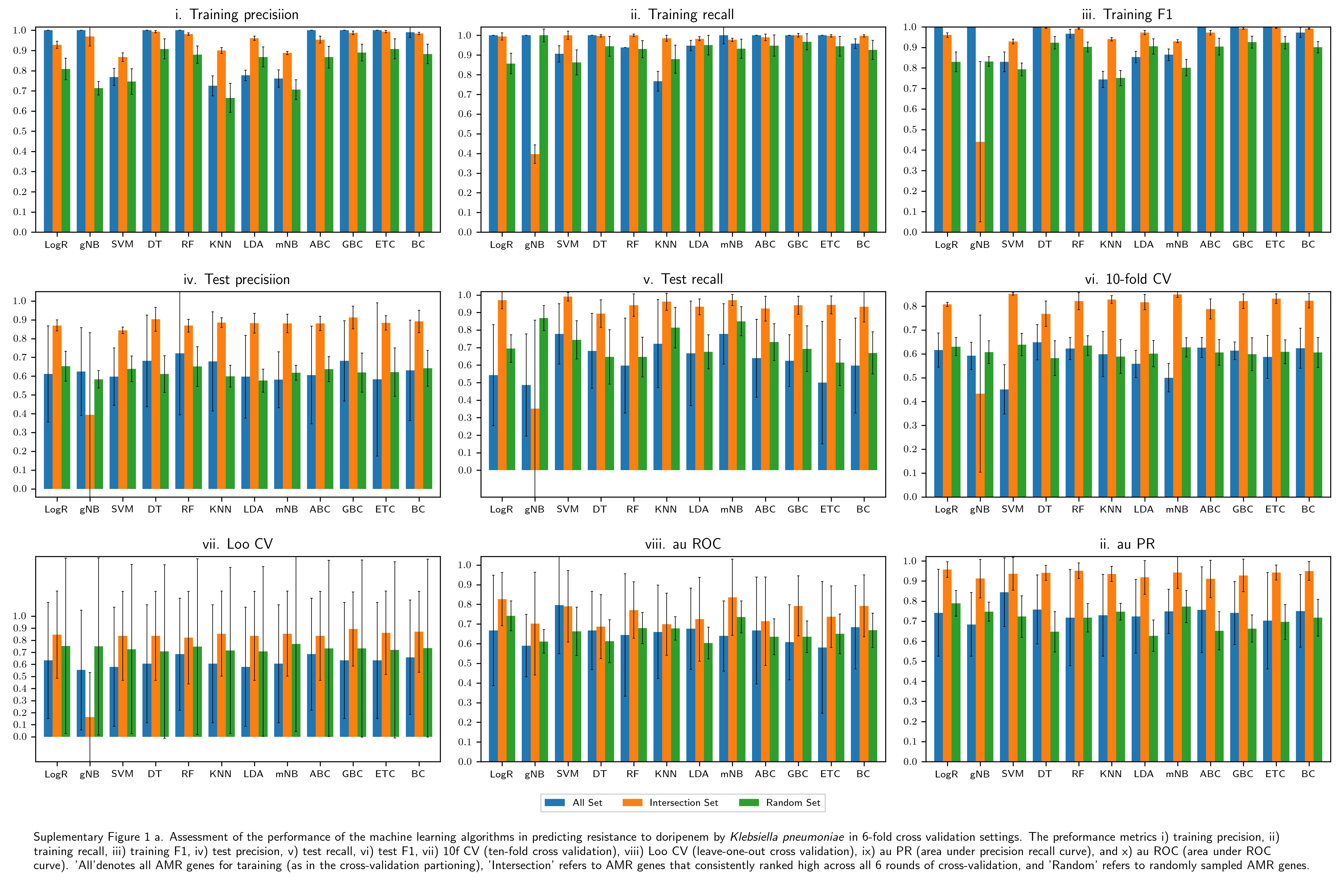

Supplement: Supplementary file 1 [file microorganisms-10-02102-s001.zip › Supplementary_Figure S1a.jpg]

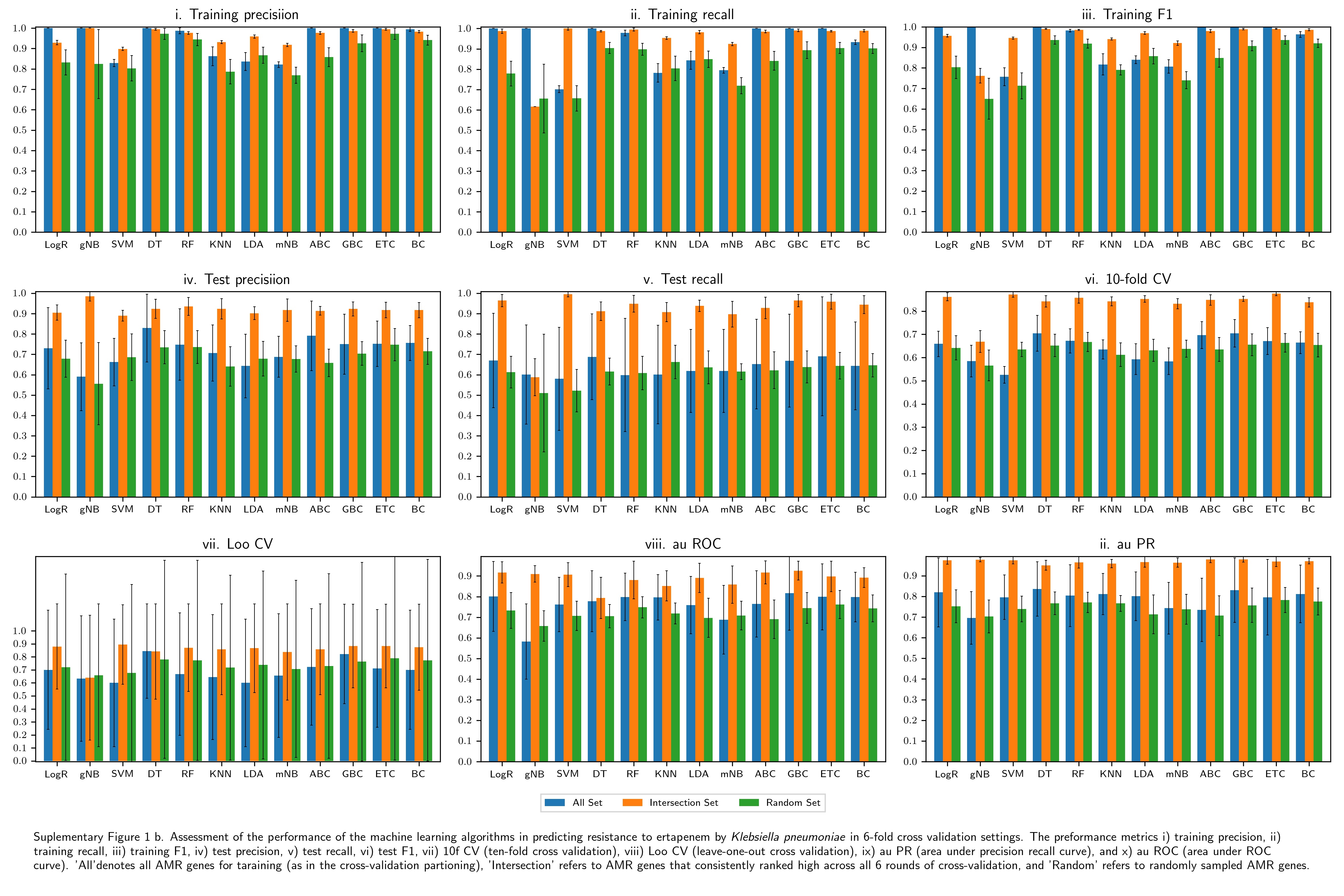

Supplement: Supplementary file 1 [file microorganisms-10-02102-s001.zip › Supplementary_Figure S1b.jpg]

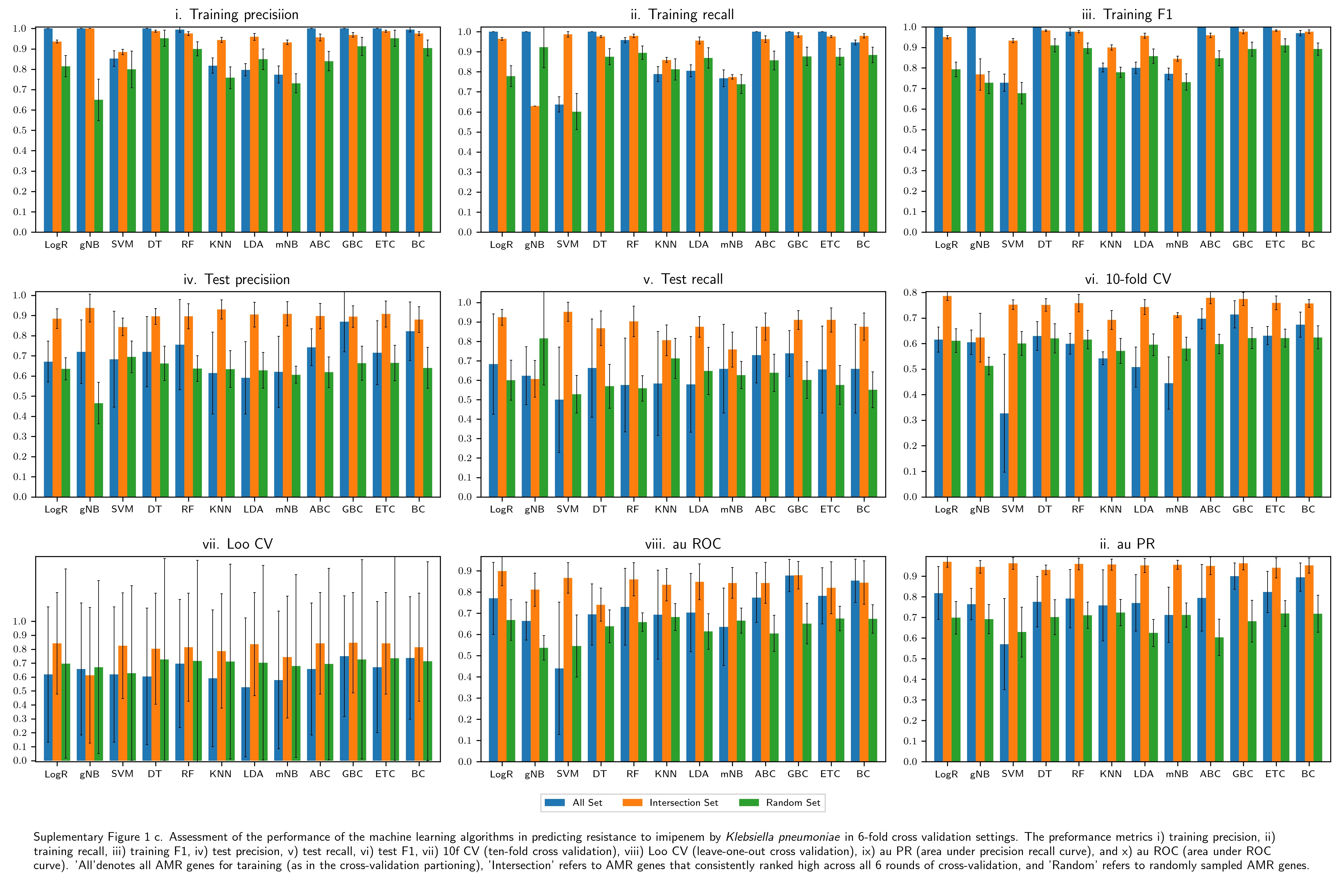

Supplement: Supplementary file 1 [file microorganisms-10-02102-s001.zip › Supplementary_Figure S1c.jpg]

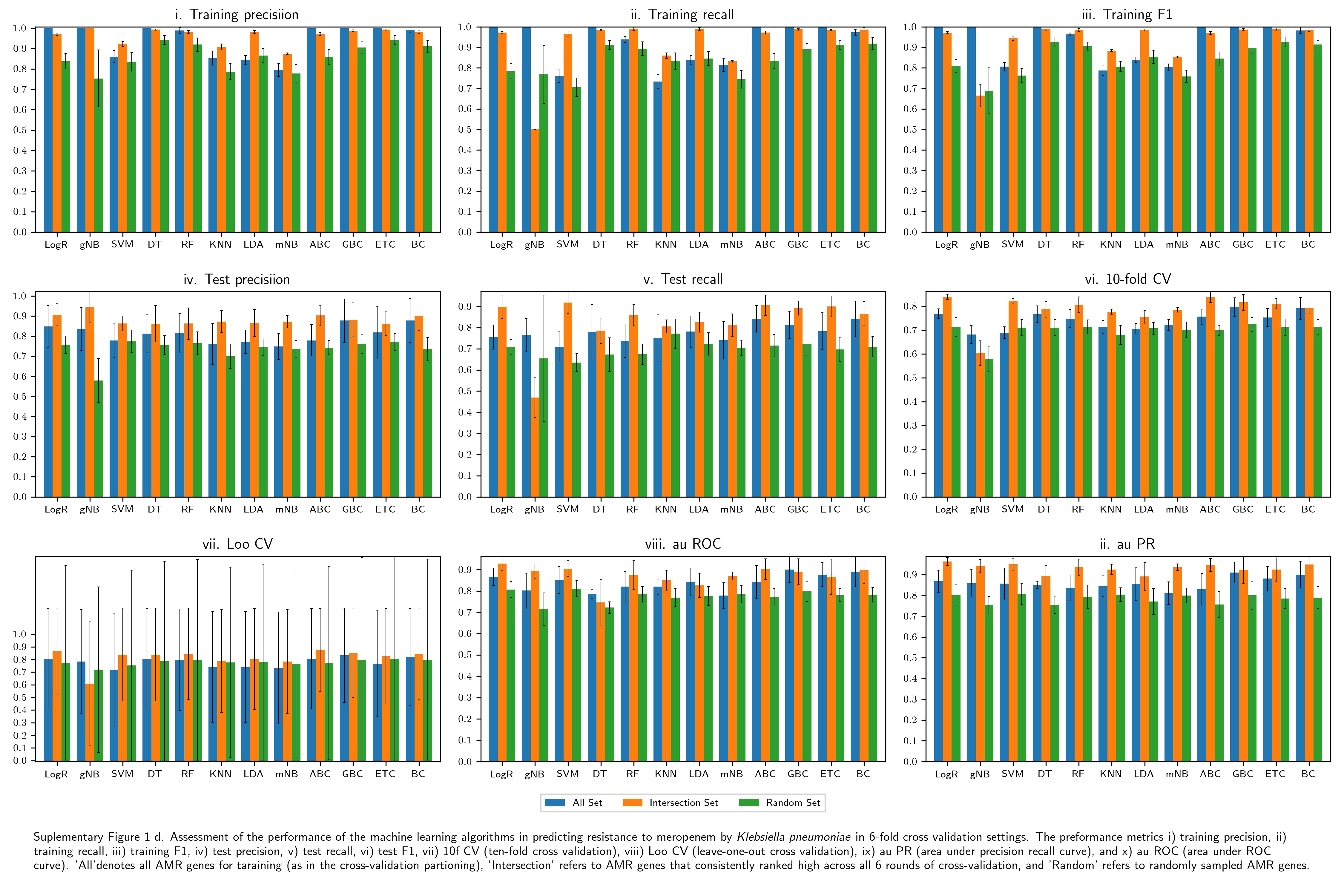

Supplement: Supplementary file 1 [file microorganisms-10-02102-s001.zip › Supplementary_Figure S1d.jpg]

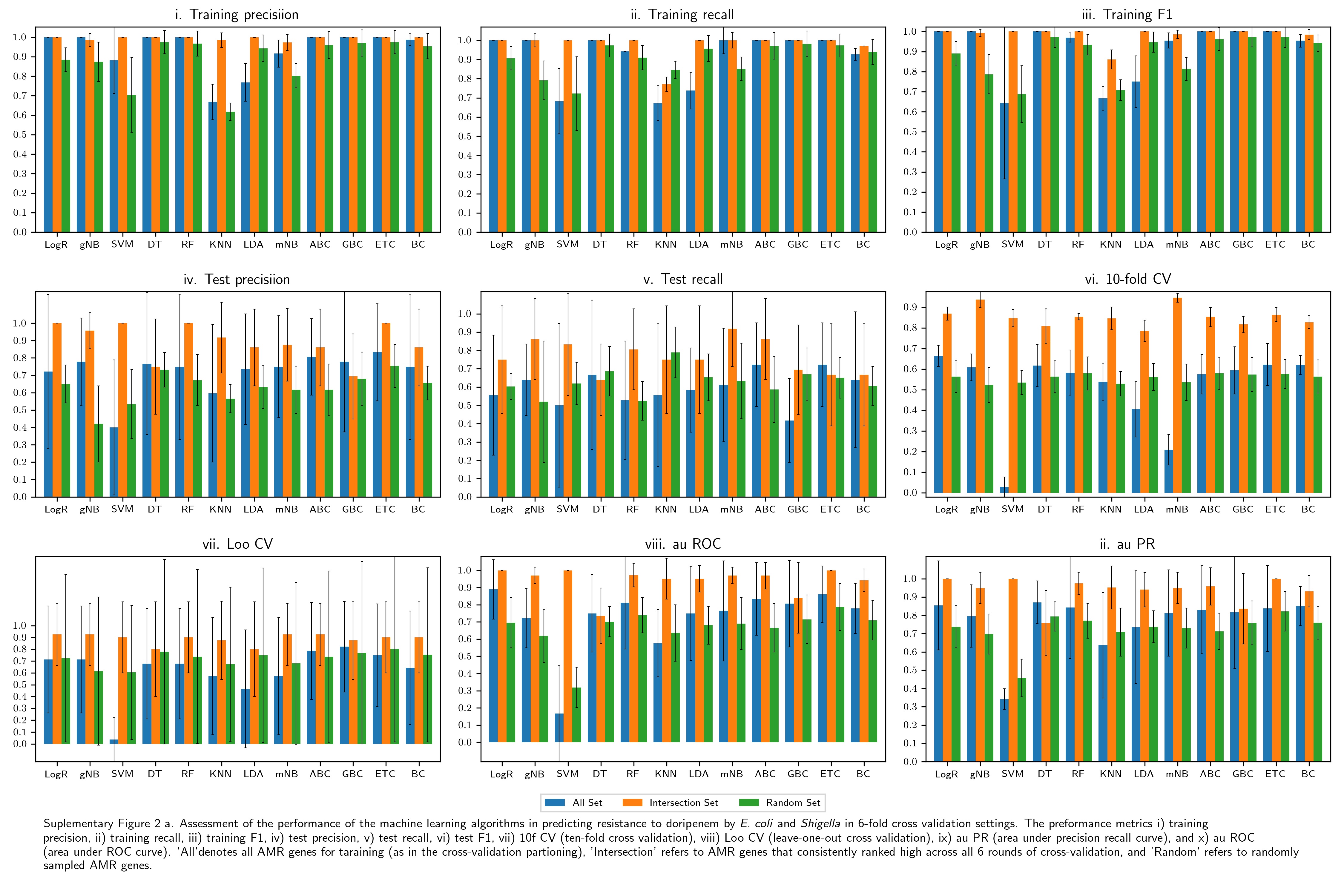

Supplement: Supplementary file 1 [file microorganisms-10-02102-s001.zip › Supplementary_Figure S2a.jpg]

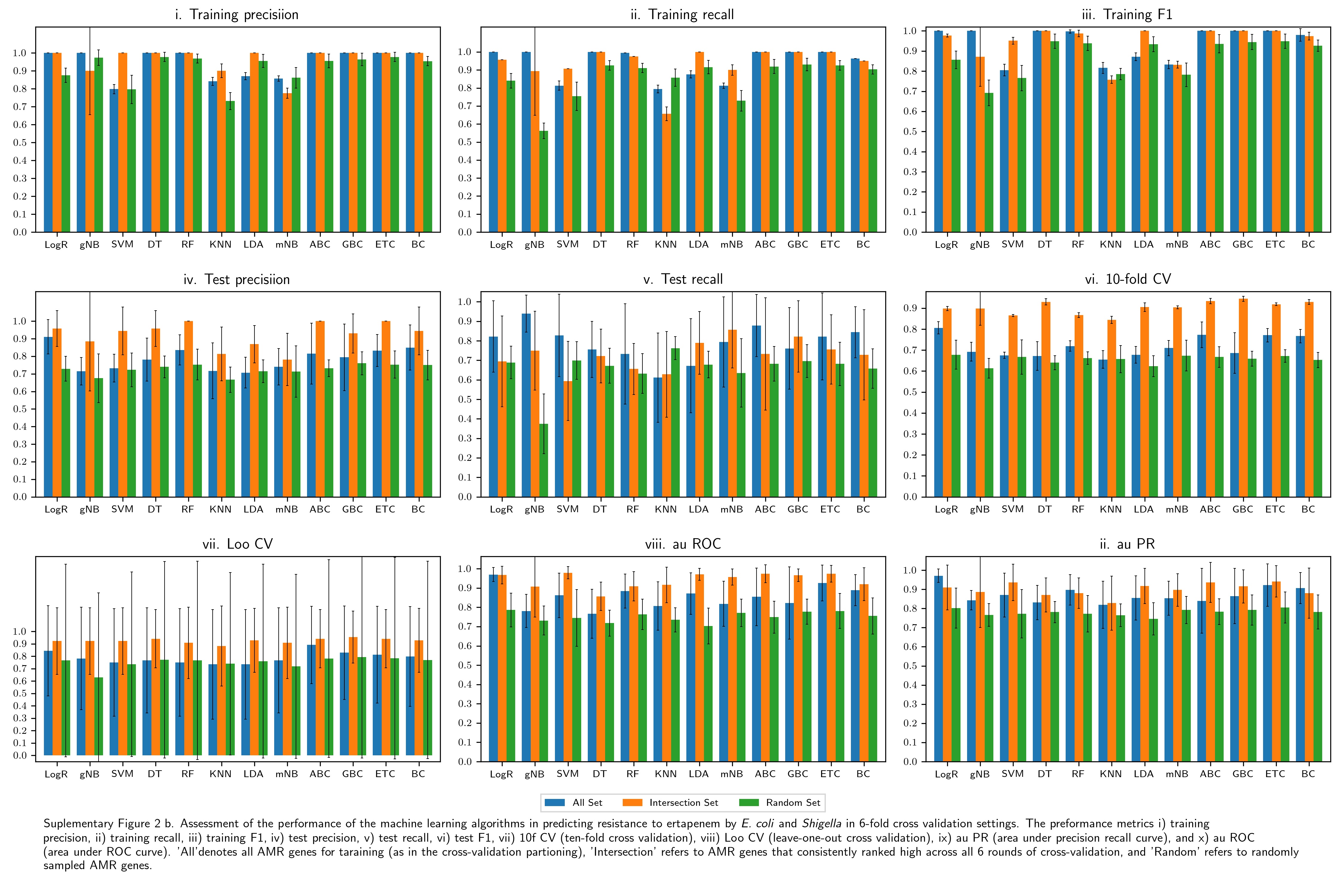

Supplement: Supplementary file 1 [file microorganisms-10-02102-s001.zip › Supplementary_Figure S2b.jpg]

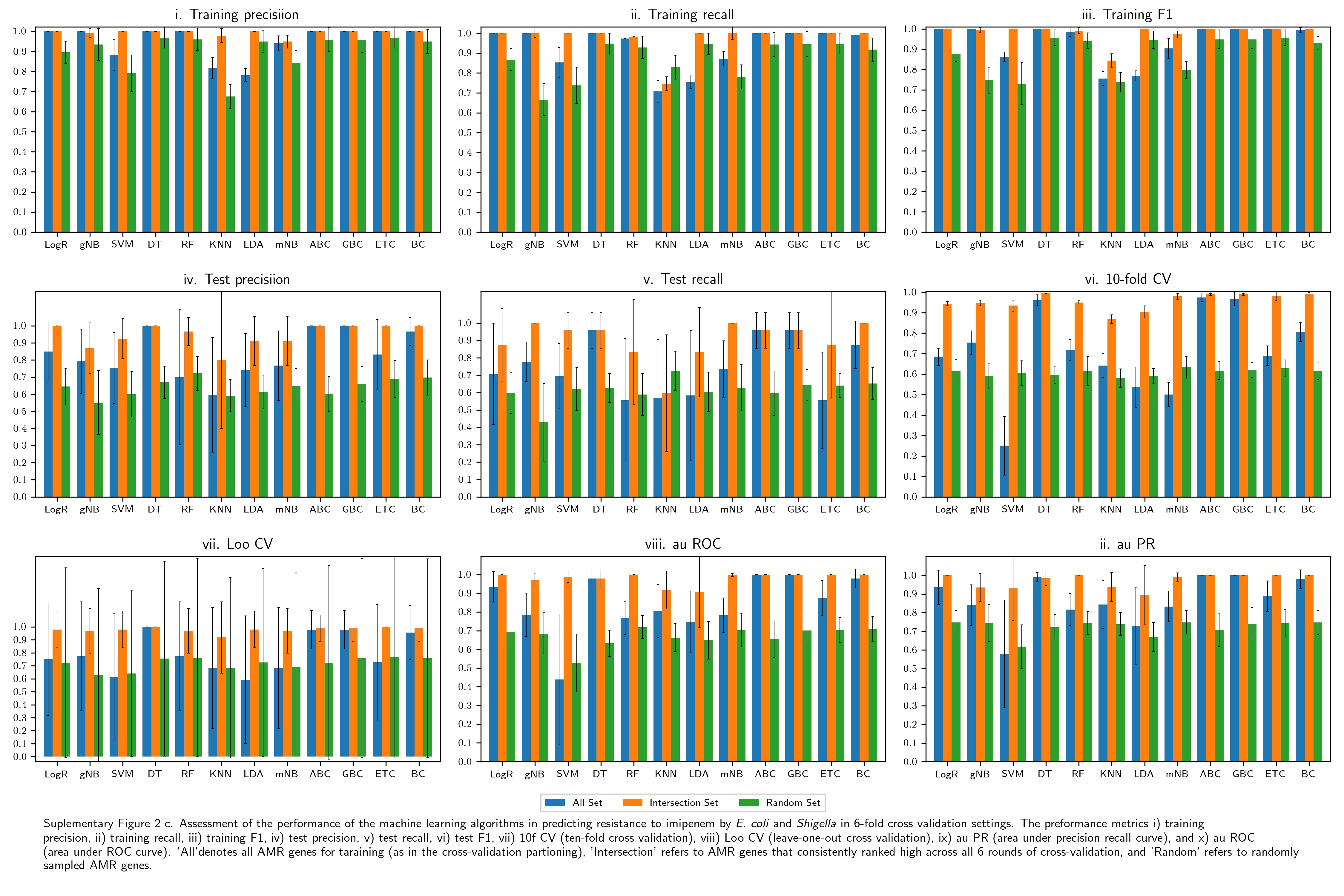

Supplement: Supplementary file 1 [file microorganisms-10-02102-s001.zip › Supplementary_Figure S2c.jpg]

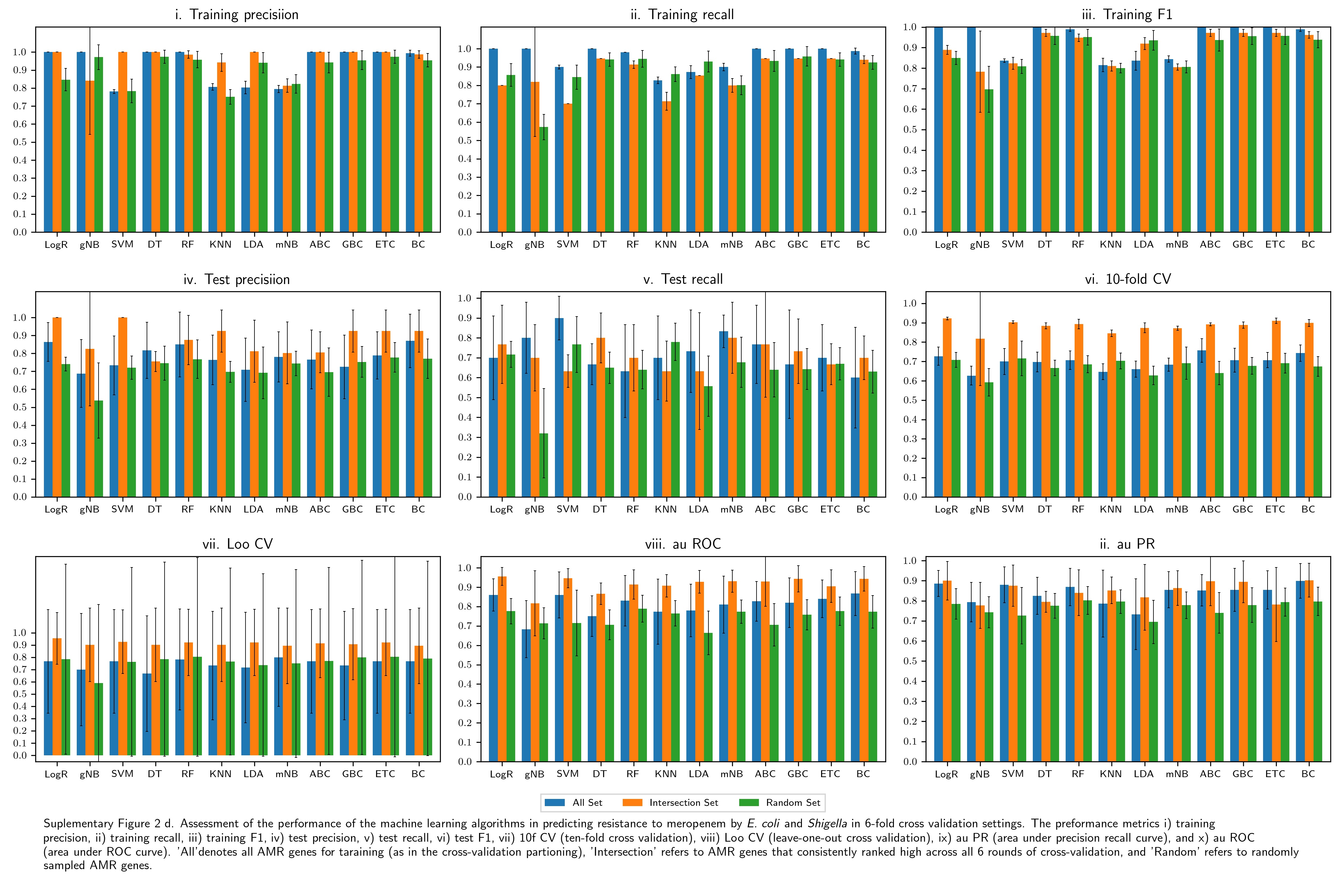

Supplement: Supplementary file 1 [file microorganisms-10-02102-s001.zip › Supplementary_Figure S2d.jpg]

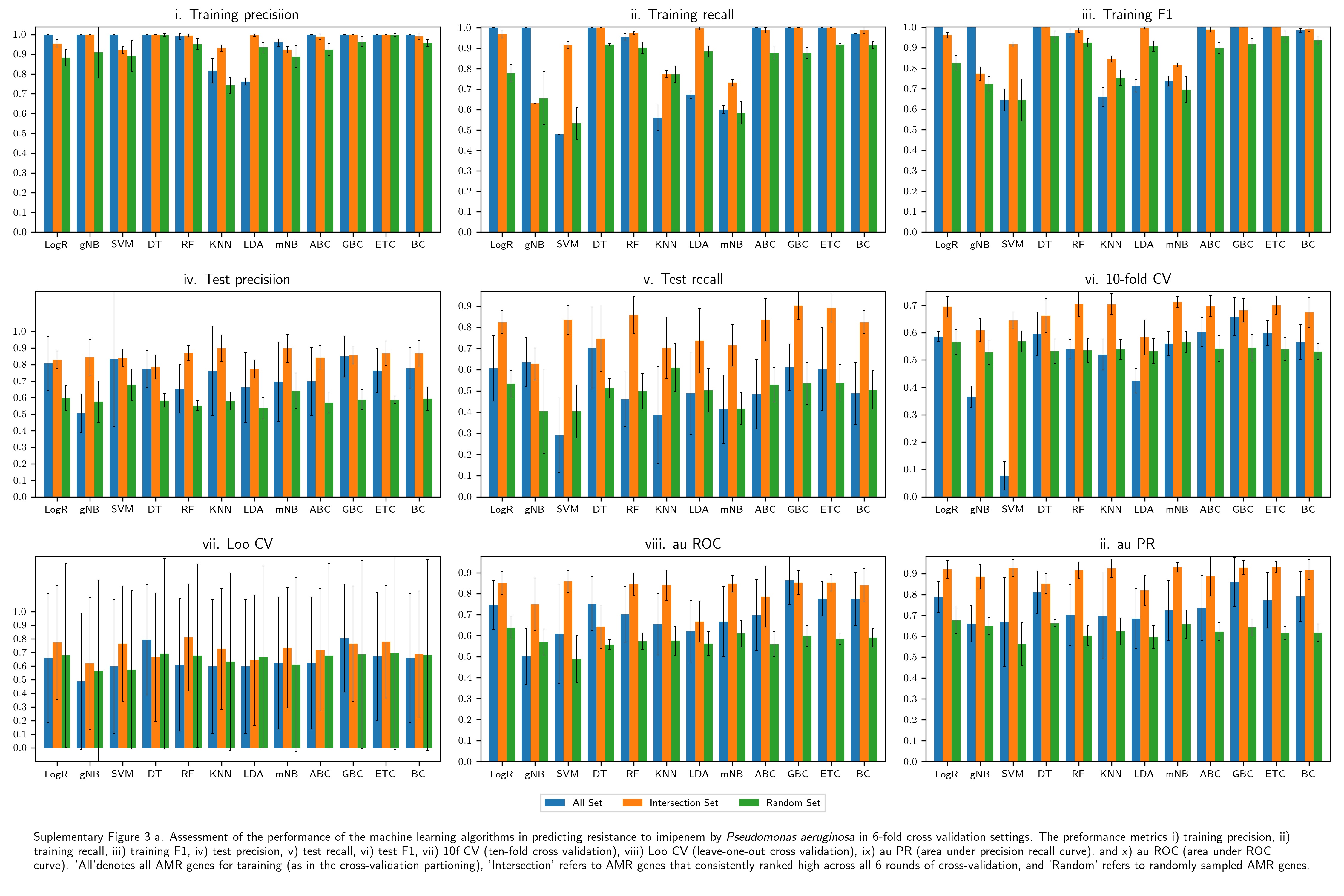

Supplement: Supplementary file 1 [file microorganisms-10-02102-s001.zip › Supplementary_Figure S3a.jpg]

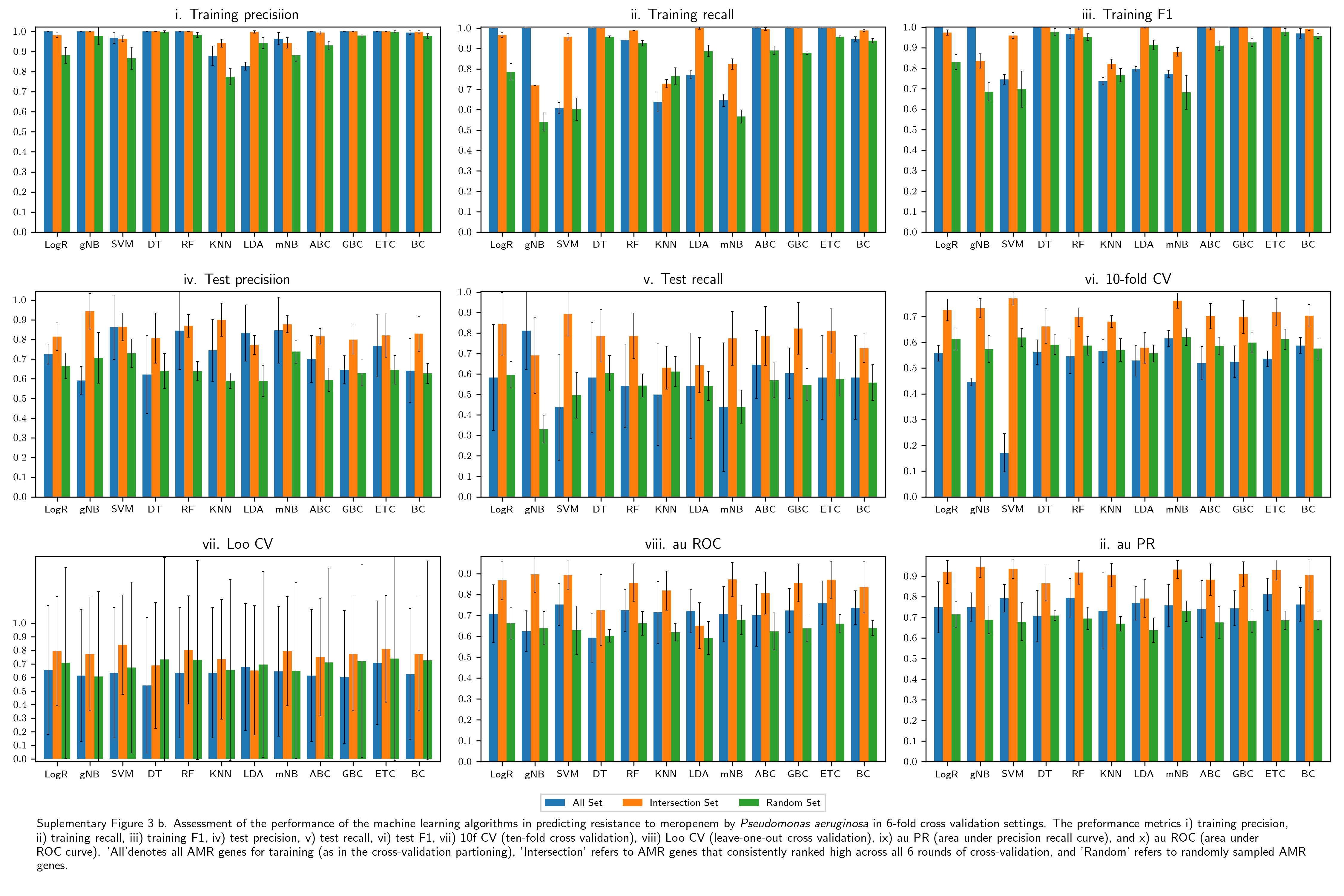

Supplement: Supplementary file 1 [file microorganisms-10-02102-s001.zip › Supplementary_Figure S3b.jpg]

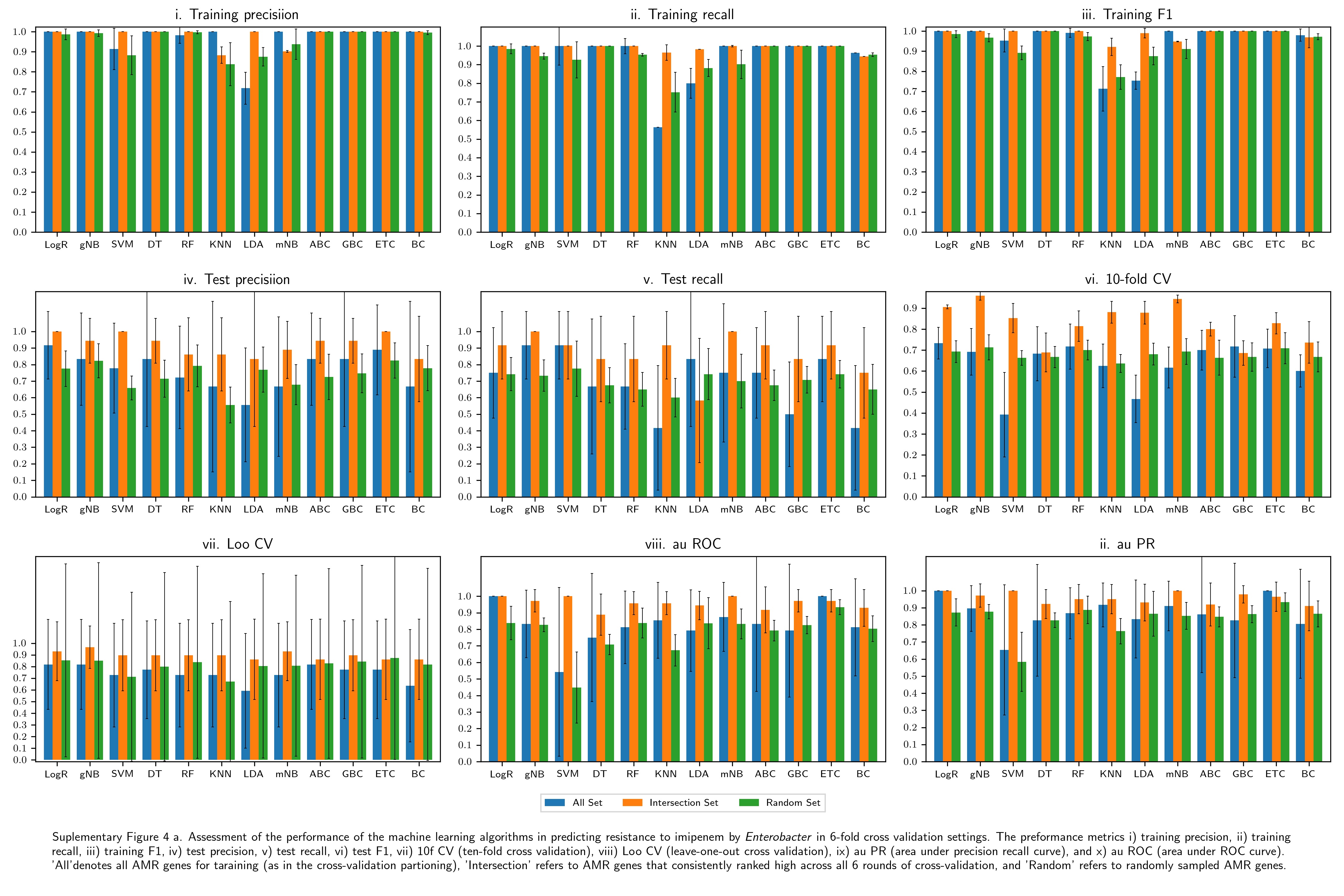

Supplement: Supplementary file 1 [file microorganisms-10-02102-s001.zip › Supplementary_Figure S4a.jpg]

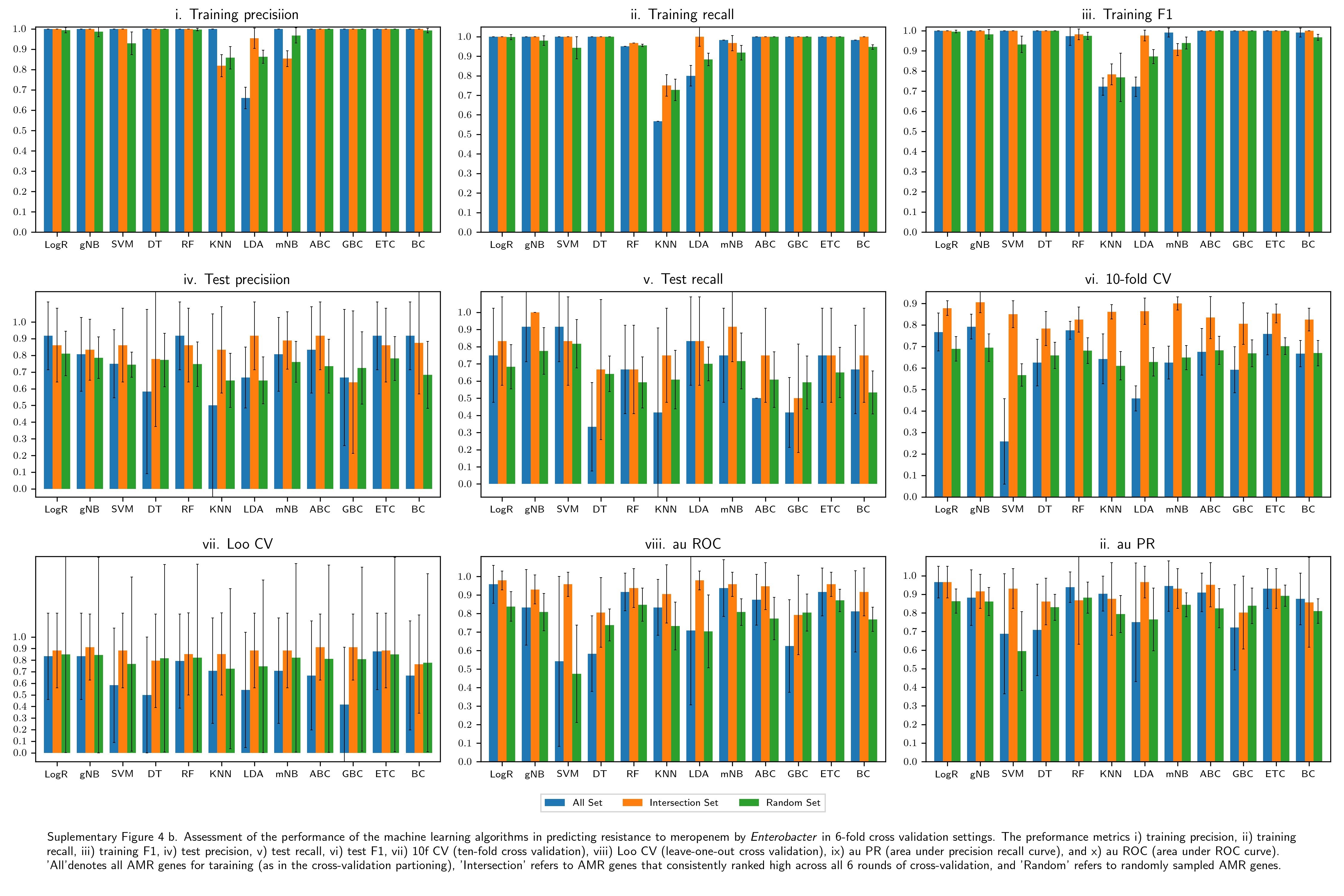

Supplement: Supplementary file 1 [file microorganisms-10-02102-s001.zip › Supplementary_Figure S4b.jpg]

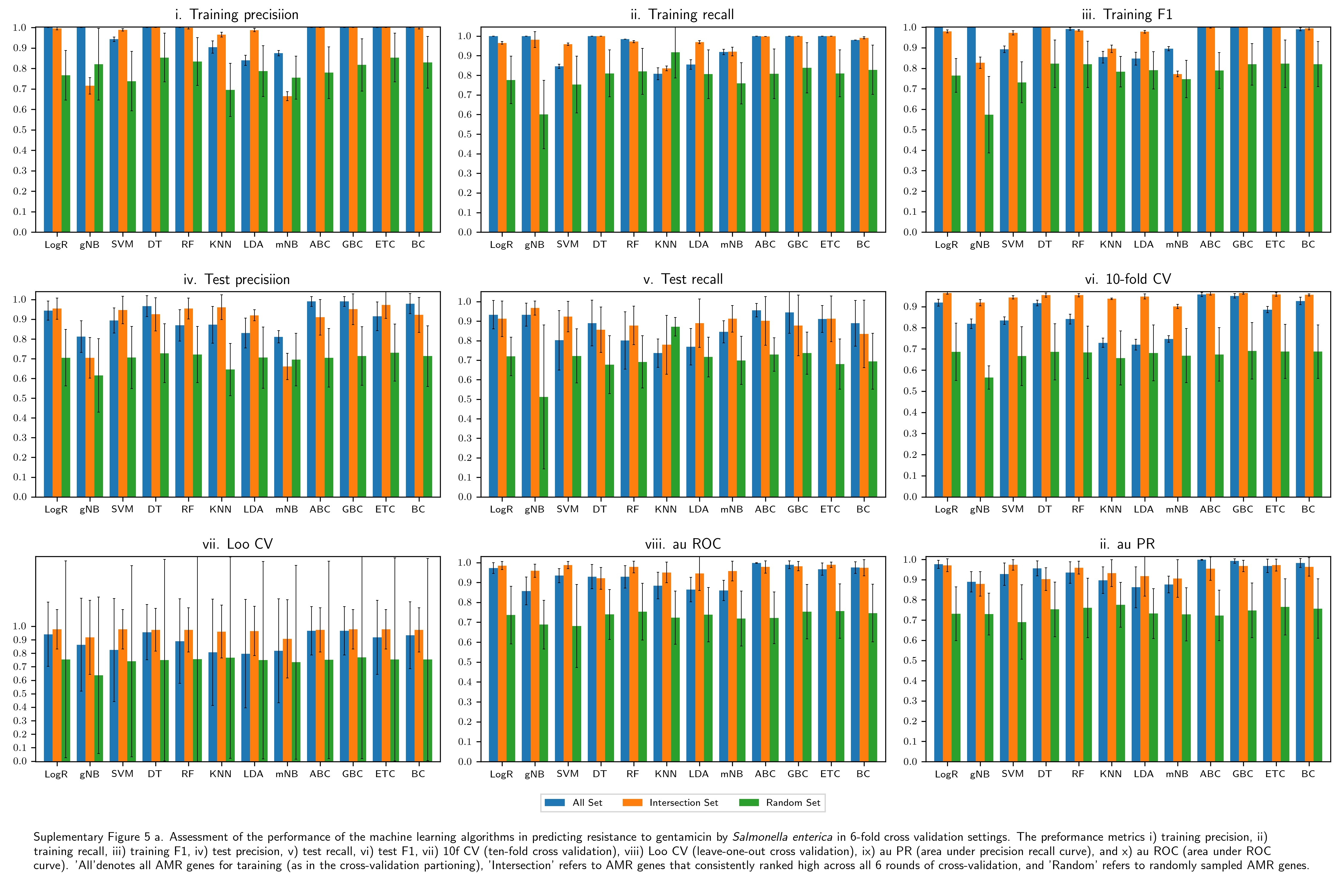

Supplement: Supplementary file 1 [file microorganisms-10-02102-s001.zip › Supplementary_Figure S5a.jpg]

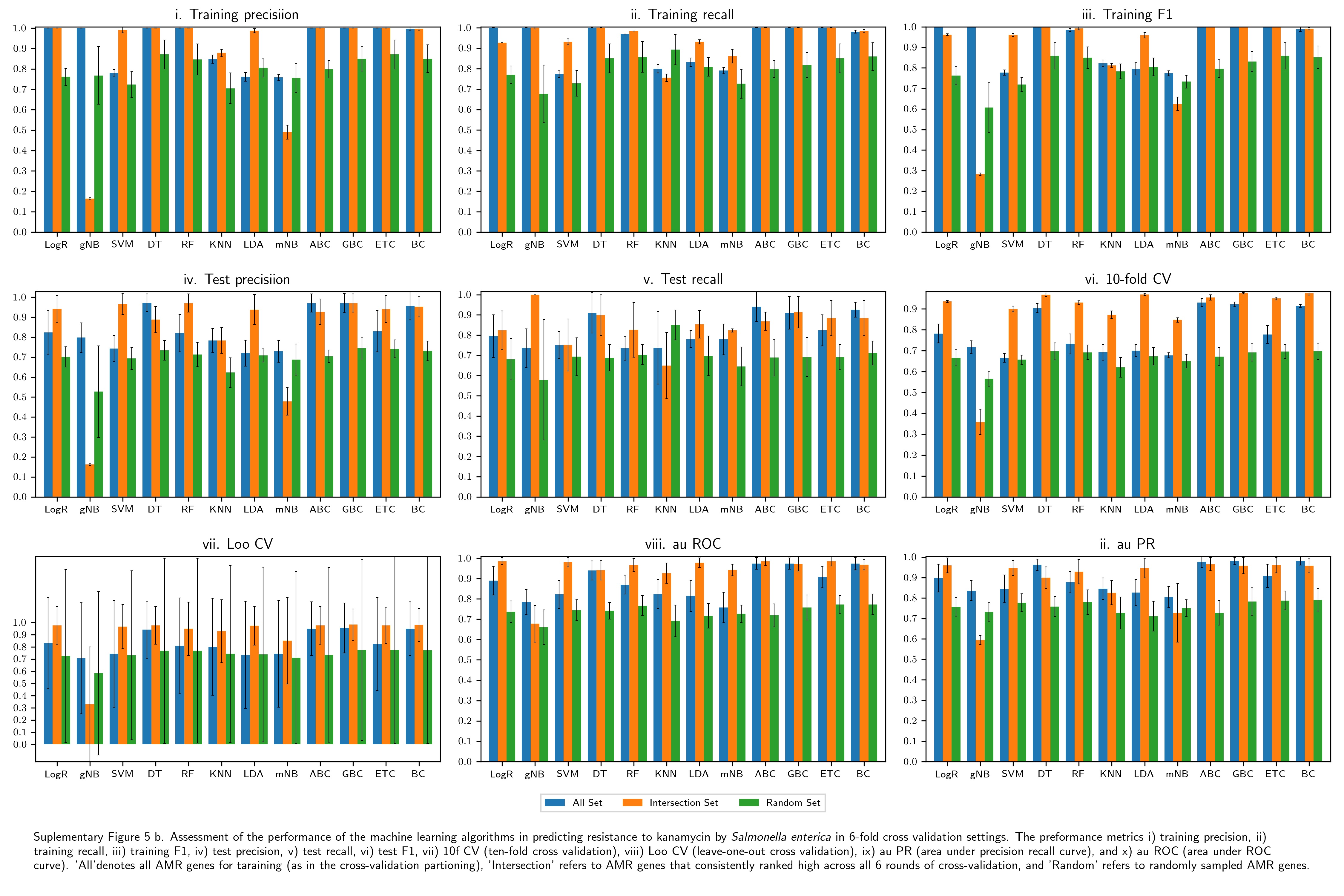

Supplement: Supplementary file 1 [file microorganisms-10-02102-s001.zip › Supplementary_Figure S5b.jpg]

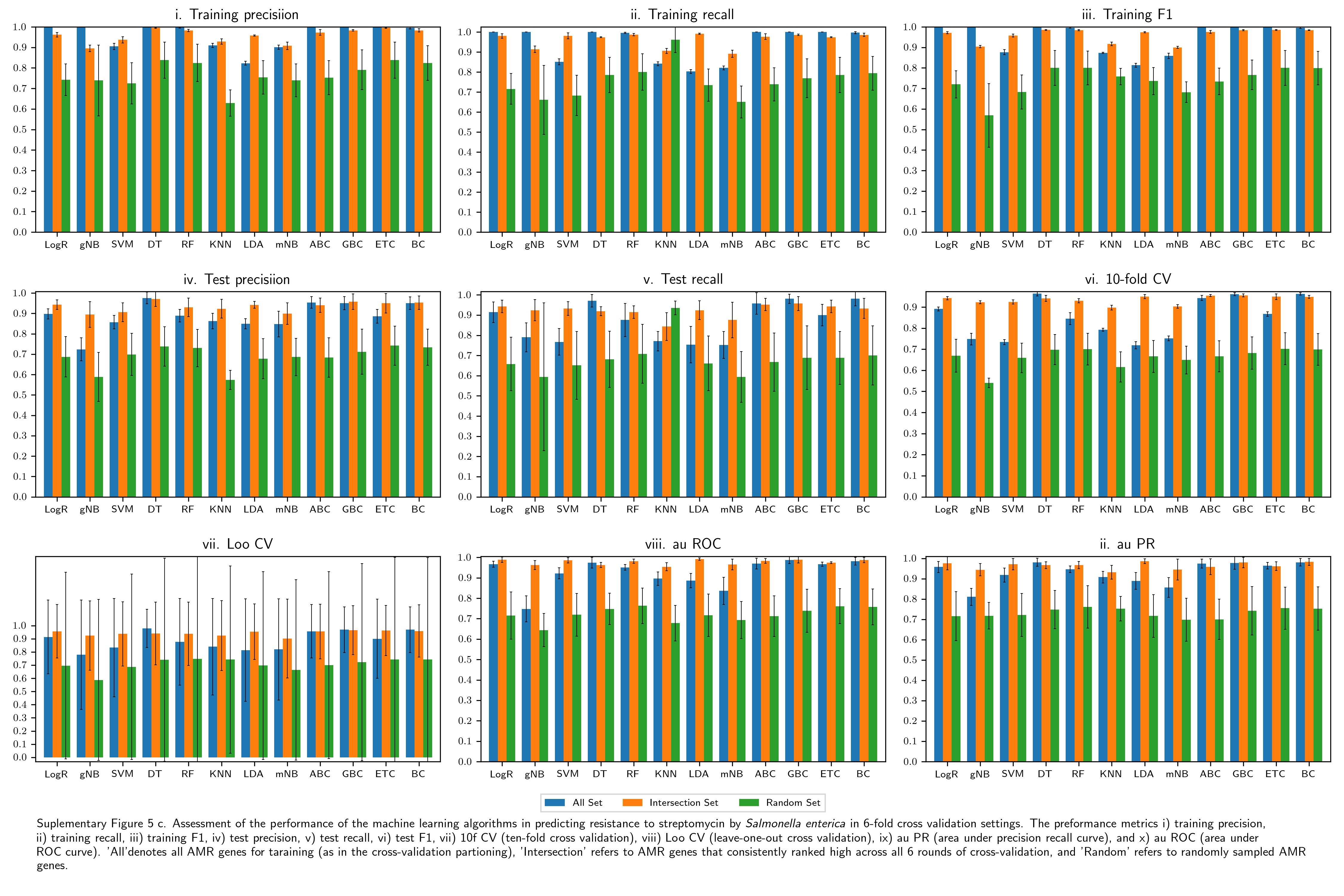

Supplement: Supplementary file 1 [file microorganisms-10-02102-s001.zip › Supplementary_Figure S5c.jpg]
